# A qualitative study of the dissemination and diffusion of innovations: bottom up experiences of senior managers in three health districts in South Africa

**DOI:** 10.1186/s12939-019-0952-z

**Published:** 2019-03-29

**Authors:** Marsha Orgill, Lucy Gilson, Wezile Chitha, Janet Michel, Ermin Erasmus, Bruno Marchal, Bronwyn Harris

**Affiliations:** 1 Health Policy and Systems Division, School of Public Health and Family Medicine, University of Cape Town, Cape Town, Western Province South Africa; 2 Department of Global Health and Development, London School of Hygiene and Tropical Medicine, London, UK; 3 Health Systems Enablement & Innovation Unit, Faculty of Health Sciences, University of the Witwatersrand, Johannesburg, South Africa; 4 Health Systems and Policy Department, Swiss Tropical and Public Health Institute, University of Basel, Basel, Switzerland; 5 Institute of Tropical Medicine, Antwerp, Belgium; 6 Centre for Health Policy, University of the Witwatersrand, Johannesburg, South Africa

**Keywords:** Innovation, Diffusion, Dissemination, Communication, District manager, Bottom up, Health system, Policy analysis

## Abstract

**Background:**

In 2012 the South African National Department of Health (SA NDoH) set out, using a top down process, to implement several innovations in eleven health districts in order to test reforms to strengthen the district health system. The process of disseminating innovations began in 2012 and senior health managers in districts were expected to drive implementation. The research explored, from a bottom up perspective, *how* efforts by the National government to disseminate and diffuse innovations were experienced by district level senior managers and *why* some dissemination efforts were more enabling than others.

**Methods:**

A multiple case study design comprising three cases was conducted. Data collection in 2012 – early 2014 included 38 interviews with provincial and district level managers as well as non- participant observation of meetings. The Greenhalgh et al. (Milbank Q 82(4):581-629, 2004) diffusion of innovations model was used to interpret dissemination and diffusion in the districts.

**Results:**

Managers valued the national Minister of Health’s role as a champion in disseminating innovations via a road show and his personal participation in an induction programme for new hospital managers. The identification of a site coordinator in each pilot site was valued as this coordinator served as a central point of connection between networks up the hierarchy and horizontally in the district. Managers leveraged their own existing social networks in the districts and created synergies between new ideas and existing working practices to enable adoption by their staff. Managers also wanted to be part of processes that decide what should be strengthened in their districts and want clarity on: (1) the benefits of new innovations (2) total funding they will receive (3) their specific role in implementation and (4) the range of stakeholders involved.

**Conclusion:**

Those driving reform processes from ‘the top’ must remember to develop well planned dissemination strategies that give lower-level managers relevant information and, as part of those strategies, provide ongoing opportunities for bottom up input into key decisions and processes. Managers in districts must be recognised as leaders of change, not only as implementers who are at the receiving end of dissemination strategies from those at the top. They are integral intermediaries between those at the at the coal face and national policies, managing long chains of dissemination and natural (often unpredictable) diffusion.

**Electronic supplementary material:**

The online version of this article (10.1186/s12939-019-0952-z) contains supplementary material, which is available to authorized users.

## Introduction

Worldwide, countries are rallying under the banner of universal health coverage (UHC). They are conceptualising, formulating and implementing waves of health reform and innovation with the aim of securing access to quality health care and financial protection for those in need [1]. It is now evident that, across countries, the road to UHC is a long-term political engagement that requires vision and commitment to building stable institutions, administrative capacity, good governance arrangements and an understanding of political economy realities when implementing reform [2]. This process requires learning from other countries and adaptation to local context, as well as marrying technical solutions with pragmatism and innovation on the ground [2, 3]. While Brazil, Russia, India, China and South Africa (the BRICS countries) face challenges in reaching UHC, including raising sufficient public health funding and meeting the demand for more human resources, it is argued that these countries must move forward as leaders in the movement for better social policies [4].

in August 2011 South Africa’s National Department of Health (SA NDoH) published a draft policy for public consultation which proposed phasing in, over a 14-year period, a range of major health reforms towards a National Health Insurance (NHI) system. This system ultimately seeks to “promote equity and efficiency so as to ensure that all South Africans have access to affordable, quality healthcare services regardless of their socio-economic status” [5]. The SA NDoH, however, recognised the importance of improving the functioning, management and quality of the country’s public health delivery system in the first five-year phase (2012–2017) before moving ahead with major health financing reform. In 2012, eleven of the country’s fifty-three health districts were named National Health Insurance pilot sites (NHI pilot sites),^1^ with at least one pilot site in each of South Africa’s nine Provinces. The overall purpose was to pilot reforms to strengthen the district health system, with a special focus on ‘Primary Health Care (PHC)^2^ re-engineering’ [4, 6, 7] and to demonstrate reforms related to the future needs of NHI implementation, for example piloting fund administration [5]. Many of the of innovations are listed in the 2011 Green Paper on NHI [5], while subsequent draft policy developments have been published in 2017 and 2018 [8, 9]. Even though no major health *financing* reform (e.g. the creation of a single fund) occurred in the first 5 years, the multiple innovations implemented in the eleven NHI pilot districts are still commonly referred to by the umbrella term ‘NHI Piloting’.^3^

The 2013 World Health Report on Universal Coverage calls for a wide variety of research studies including research on detection, treatment and diagnosis to health policy and systems research acknowledging the importance of local knowledge to answering UHC research questions [10, 11]. A bottom up approach to understanding health systems recognises that multiple actors are engaged in the politics of health system change at the coal face, and the need for more policy analysis and health systems research in Low- and Middle-Income Countries (LMICs) to understand this politics of implementation and change has been identified [12–16]. McIntyre and Klugman [17] note that most literature on health system reform focuses only on structural and technical issues. They call for a research focus on ‘the human face of decentralisation’ to understand the software issues affecting managers and front-line workers (being and feeling part of the process of policy development and receiving early communication). Health managers are a key ‘human face’, going through processes of collective sensemaking - “the way managers understand and interpret and create sense for themselves based on information surrounding the change” - and then a process of sense-giving - “their attempts to influence the outcome, to communicate their thoughts about the change to others, and to gain their support” [18].

District management teams (DHMTs) are subject to and are part of the politics of implementation and change. They must “mix and allocate the available resources in the best possible way to meet the basic health needs of the community they serve” [19] and in policy reform processes must also “interpret and implement what is required” [20] from top down policy instructions.

Seeking to contribute to understanding the human face (specifically managers) of change in South Africa, this research explored district-level senior manager experiences of the dissemination (by the National government) and diffusion of innovations (in the NHI pilot sites) in the early period of introduction (2012 - early 2014).

## Conceptual framing

Greenhalgh et al. [21] offer a lens to interpret the dissemination and diffusion of innovations in South Africa’s NHI pilot sites. They define innovation in service delivery and organization as a “novel set of behaviours, routines, and ways of working that are directed at improving health outcomes, administrative efficiency, cost effectiveness, or users’ experience and that are implemented by planned and coordinated actions” [21]. An innovation can be old in one context, but completely new in a different context. Greenhalgh et al. [21] acknowledge that innovations that include new behaviours, routines and ways of working must be disseminated, diffused, adopted, implemented and sustained in complex systems over time to make a difference. To help make sense of these multiple components, building on Rogers [22] work, these authors [21] developed a comprehensive conceptual model, through systematic review, that depicts determinants of diffusion, dissemination and implementation of innovations in health service delivery and organisation to help make sense of complex situations.

**Fig. 1:**
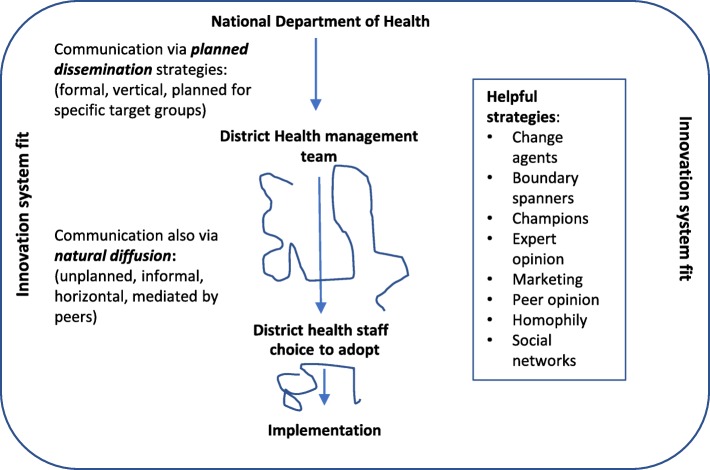
Communicating top down innovations: dissemination and diffusion Source: key concepts are taken from Greenhalgh et al. [21] conceptual model for considering the Determinants of Diffusion, Dissemination, and Implementation of Innovations

The conceptual model is comprehensive and includes multiple components. Dissemination and diffusion are concepts identified under the ‘communication and influence’ component of the model. Understood as a continuum, at one end dissemination strategies are formal, planned efforts to persuade target groups to adopt an innovation (often centralised and occurring through vertical hierarchies), whereas, at the other end, in pure diffusion, the spread of innovations is unplanned, informal, decentralised, and largely horizontal or mediated by peers, see Fig. 1 [21]. Effective communication is also recognised as a key component of implementation success by both top down and bottom up policy theorists [23–26].

It is possible for those driving policy change to influence actors to adopt an innovation by using strategies that have proven to help disseminate and diffuse reforms [21]. These include taking account of the structure and quality of actors’ *social networks*: horizontal networks can be effective for spreading peer influence and reframing meaning, while vertical networks can be effective for cascading information and authoritative decisions downward. Second, adoption is more likely if individuals are *homophilous*, sharing certain common traits such as education and professional backgrounds. Third, expert *opinion leaders* can influence the beliefs and actions of others through their authority and status, while peer opinion leaders ‘exert influence through their representativeness and credibility’. Opinion leaders, however, have to find the innovation appealing and buy into it; they could sway opinion either way. Fourth, the use of *champions*, key individuals in a social network who can garner support from others, can make adoption more likely. Fifth, the use of *boundary spanners* – people who have significant social ties inside and outside the organisation –can help to link the organisation to the outside world with regards to an innovation. Actors in these various roles can serve as intermediaries to promote adoption and implementation [22]. Finally, the use of *formal planned dissemination strategies* that target particular audiences with appropriate messaging and appropriate communication channels can facilitate innovation diffusion [21]. Another key factor affecting adoption is ‘*innovation-system-fit’*, the interplay between the innovation and the context in which it is rolled out, policy analysis theory also recognises the critical role of context. In the Greenhalgh et al. model implementation takes place after the adoption decision [21].

These concepts and strategies from the model provide us with a lens to identify dissemination strategies and diffusion in the sites in the early stages of change. A bottom up perspective explicitly draws on the experiences of district level senior managers working in and managing the day to day functioning of the health district [27].

## Methods

### The aim of the study

The research specifically sought to understand, from a bottom up perspective, *how* efforts by the National government to disseminate and diffuse NHI piloting innovations were experienced by district level senior managers, in order to draw key lessons from the experience on practices that enable the diffusion and dissemination of innovations in ways that are helpful for health managers at the coal face.

### Setting

In 1994, the South African government inherited a fragmented and regressive health system that was set up to serve a minority of the population with a hospital based curative focus [28]. Embodying a vision to establish an equitable health system with a PHC focus, the government developed a National Health Plan for South Africa in 1994 which laid the foundation for establishment of a district health system [29].

South Africa has a fiscal federal system of government which decentralises authority for a range of powers, functions and budgeting from National to Provincial and Local government. National government is the main revenue collector and financial transfers are made via equitable share allocations (general-purpose) and conditional grants (specific purpose) to provincial and local governments to provide and finance services in the different sectors (including health) [30]. The National Department of Health (SA NDoH) is primarily responsible for policy making, setting standards and regulations while the Provincial Departments of Health are responsible for the provision and financing of public health care services relying largely on the National government for financial resources. Local municipalities are responsible for environmental health services, and PHC services are delivered through the district health system (DHS), which is by design a lower level of the provincial health authority [30]. The Provincial government determines the amount of decision space granted to each DHS, including financial and human resource authority. The DHS is led by a district manager (DM) who works together with programme, hospital and support service managers as a district management team; across the fifty-three health districts, the structure of district management teams vary in practice [31]. In South Africa, management competence in facilities and districts at all levels is varied with managers having a diversity of backgrounds [32]. Today South Africa has fifty-three health districts established through the 2003 National Health Act. The district health system is still being institutionalised in many ways and whilst it has achieved gains, the current challenges include different interpretations by Provinces on what constitutes the most appropriate structure for a DHS, sufficient delegation of powers to managers in districts, that formal accountability mechanisms are not always in place, shortages of human resources and district hospitals that are sometimes poorly coordinated with PHC services [33].

### Study design

Context-sensitive and flexible approaches are needed to understand and support evaluation of large scale health programmes due to the longitudinal and complex nature of implementation [34]. We employed a case study design, appropriate for research of contemporary phenomena in complex health systems where events and experiences of change emerge while research is undertaken, the phenomena being directly influenced by the context [35]. More specifically, a multiple case study design allowed for deeper explanation of the experience through more than one case [36].

We defined the case as the experience of district level senior managers of the dissemination and diffusion of innovations in the period 2012 - early 2014. We explored this case in three district sites, each district thus serving as a case study.

### Site selection

Three sites were selected from the 11 NHI pilot districts. Selection criteria included (1) a district that was actively receiving information from other levels of government and/or was implementing some of the innovations, (2) access to the site, meaning district managers were prepared to give us access to staff and (3) rural/urban mix to capture variation in experiences of implementation possibly linked to geography.

### The study sites

To maintain anonymity, only general information about the study site is given to provide context. The sites were underperforming relative to other districts in the country. For example, in 2013/14, the sites had facility maternal mortality ratios and an incidence of under 5 severe acute malnutrition higher than the national average [37]. The sites are nested in geographic areas where health and social services were hugely under-resourced and neglected during the apartheid era pre-1994 [38].

### Data sources and data collection

As a first step, we conducted interviews in late 2012 – early 2013 with 7 provincial level managers who played a role in information sharing and/or rolling out NHI piloting, to help purposively select the three district study sites. Guides included questions relevant to early dissemination efforts between the three levels of government, leadership for NHI piloting and information on roll out to the districts. While the paper focuses on the experiences of senior managers at district level, information from provincial interviews were relevant to contextualizing experiences and were thus included in analysis.

Between September 2013 and July 2014, we undertook 2 site visits to each district, interviewing 31 members of DHMTs. Participant selection criteria included (1) being a member of the DMT (a senior manager) and (2) involvement in the dissemination of and/or implementation of NHI piloting innovations in the district. We also attended meetings as non-participant observers. Recruitment started when we presented the research protocol to the district manager in each site for approval, needed to conduct the research. At this time, we also requested the district manager to identify management team members who met the selection criteria as interviewees. In interviews with these managers we also asked them for their ideas about prospective participants to reduce selection bias. Each prospective participant was e-mailed an information letter about the project as well as a consent form and both were discussed before the interview. The semi-structured interview guide included questions that would allow us to explore the respondents’ experience of the process, including personal understandings of the vision and goals of NHI piloting, key activities taking place in the district around NHI piloting (including early communication), key activities and assumptions driving the dissemination of these activities as well as individual feelings about involvement in the process of change (individual roles, responsibilities and relationships with others) – all from the perspective of the managers themselves, not from the policy documents. Since multiple theories of change may co-exist in processes of reform, we included questions seeking to elicit assumptions and gather information on NHI piloting from the perspective of managers. We also included prompts in the interview guide from the Greenhalgh et al. [21] dissemination strategies (e.g. the role of networks, champions etc.) to help identify dissemination strategies and any emergent diffusion processes that managers were exposed to. The use of theory and thick description supports the transferability of lessons beyond the cases [36].

### Data analysis

Cross case analysis helps to deepen understanding and explanation beyond that which a single case study can provide [36]. Cross case analysis allowed the patterns and underlying explanations to be compared, supporting transferability across sites [36].

In each site, all interviews were transcribed verbatim. The first author led the process of analysis. The first author developed a deductive coding matrix in table format, using the strategies for diffusion and dissemination identified in the Greenhalgh model [21] as headings to support data extraction and analysis across the three sites [36]. A code book was developed to ensure each researcher understood each deductive code. Allowance was made in the deductive table for inductive coding to capture any emergent findings and ideas. In each site, data were manually extracted from each interview into the deductive coding matrix by a site research team.

The completed matrix from each site was reviewed by the first author to identify key themes and explanations related to manager experiences of diffusion and dissemination in each site. For each site an initial story of what factors were enabling or constraining national government efforts was developed, this was done in consultation with site research teams to promote rigour in the analysis. The lead author then identified key similarities and differences across the three sites for inductively developed themes that helped to answer our research question.

The lead author, in a more deductive approach, also drew on the Greenhalgh et al. [21] strategies to look for patterns in each site, specifically grouping codes into named patterns of ‘strategies and actors related to dissemination’ and ‘strategies and actors related to diffusion’, as well as looking for any factors related to the context (innovations system fit) that may have enabled (or not) the diffusion and dissemination of innovations in that site. These patterns were discussed and verified with the research team. Parts of this research was also fed back to managers from the three sites in a one-day feedback session, this provided some opportunity for member checking. The findings of this paper will be developed in to a policy brief as well as incorporated into our teaching, which includes many students working in the health system in South Africa.

Draft cross case findings were written up by the first author and reviewed in iterative rounds by researchers across the three sites until a final synthesis was reached. The process of writing was also a source of rigour as co-authors were able to verify the lead author’s synthesis as it evolved.

The first author also reflected on top down and bottom up implementation theory [39] to situate the findings within the broader government context within which dissemination and diffusion was taking place.

## Results

The results section presents themes that emerged on how efforts by the National government to disseminate and diffuse reforms were experienced by district level senior managers and *why* some dissemination efforts were more enabling than others in the process of adopting reforms. Insights into senior managers’ own subsequent roles in the diffusion and dissemination process are also shown. A brief timeline of events is presented first.

### The policy

NHI pilot sites were selected as pilot sites by the SA NDoH, rather than by provincial departments of health. Managers and staff in some sites only heard they were a pilot site via media announcements. The SA NDoH had set aside R150 million for the 2012/2013 financial year to support work in the pilot sites to test innovations for future NHI implementation and to strengthen the health system [40]. In 2012 innovations included but were not limited to PHC re-engineering, including (1) the contracting in of private general practitioners into public clinics; (2) the introduction of district clinical specialist teams (DCSTs)^4^ (3) management capacity building and (4) strengthening of maternal referral pathways [6, 41]. Roll out of innovations took a different pace in each pilot district. Some innovations, for example Municipal Ward Based Outreach Teams, were also being introduced in other health districts (non-pilot sites) in South Africa at the same time.

Of the R150 million, each NHI pilot district was intended to receive a conditional grant of R11.5 million (approximately $800000) from the National government as a resource beyond the normal budget allocations they received from their Provincial budgetary allocation [42]. These grants were to be allocated to fund NHI business plans that captured activities and outcomes for each NHI pilot site. “District NHI Business Plans provide an opportunity for ‘bottom up’ learning and experience to inform central NHI-related policy and the roll out of reforms to other districts” [6]. Due to the large number of reforms that were envisioned [5], it was not clear to the research team which innovations were high priority at the outset or how innovations would be selected for a particular annual business plan in each district and there was no clearly outlined monitoring framework. Strengthening of the district health systems and PHC was however a clear focus area. It was also not clear how the NHI Business Plan would resonate with or advance the goals embodied in the district health plan, developed separately.

### Engaging with senior managers to institutionalise reform

The conditional grant and the need to develop a district level NHI Business Plan (annually) were key mechanisms that drove district managers’ active engagement with the piloting process. In the beginning, key sources of frustration included lack of clarity on the amount of money that would be received, how it would be paid (which ultimately had consequences for annual budgetary cycles) and a feeling that the SA NDoH objectives for NHI piloting which guided the development of the business plans were not well aligned to actual district needs (and nor, sometimes, were provincial objectives). Initially, all districts had prioritised basic operational requirements in their Business Plans; for example, equipping under-resourced facilities, providing office chairs and desks for new district-level staff members, as well as general infrastructure development and maintenance at facilities (some of these were needed to facilitate space and accommodation for new cadres at facilities that were part of NHI piloting innovations). For the districts, these ‘basics’ were important preconditions for the implementation of the broader reforms and managers felt a top down approach compromised local level planning. The initial confusion resulted in feelings of rejection in the districts;

*“I’m not quite clear as to what informs certain focus areas to be decided upon [In the NHI Business Plan] (…) To me at the moment, and I could be wrong, it looks like, these [business plan] focus areas were decided upon by national. (…) There is a need to bring in a bit of flexibility, into how we should look at the plan. (…) So even if, as a district you realise that you’ve got certain priority areas that you need to attend to, you have to shelf them … and we were only allocated RXXX million, … you understand? So, it’s like, you’ve been restricted access to the wallet*” (SM2, Site 2).

Key concerns centred on the limited decision-making authority at district level and the need to apply for funds from the SA NDoH rather than receiving the full conditional grant at the beginning of the cycle. In one district, delays in capturing expenditure on the system was perceived as a lack of ability to spend. A provincial manager did however note that within limits, equitable share allocations^5^ were also useful in supporting roll out.

After initial confusion, a Monitoring and Evaluation (M&E) team^6^ set up by the SA NDoH travelled to pilot sites and disseminated information on rules and guidelines for developing the business plan and spending the conditional grant. After some time, the M & E team facilitated upward negotiation for some flexibility/adaptation in the development of the plans allowing for prioritisation of the basics. Concerns were allayed over time:

“*Remember we talked GP contracting because we can’t talk GP contracting without being ready in terms of proper accommodation in our facilities, you know! So that’s why we started to say we want to buy equipment [basics]. And they saw… . I’ll show you. We have the report and everything in terms of the equipment that we bought. Now all our clinics, you can’t believe it, we have built them with this basic equipment, even the IT. There is no clinic here without a computer now.*” (SM2, Site 1).

An NHI coordinator identified three key factors that enabled the work of the M & E team: a willingness to talk; swift responses to communication requests; and quick turnaround times with decisions as they had power to take decisions (it was only when a request had to be signed off by much higher channels that this added to the turnaround time).

### Dissemination of information

In 2012, the Minister embarked on a major dissemination event, a roadshow engaging with a range of stakeholders in all the NHI pilot districts, including disseminating information on NHI piloting and gathering information for the piloting. This involved over 15,300 stakeholders [6], and the National government also produced leaflets and booklets explaining the NHI in multiple languages [43, 44]. The roadshow events were publicised through government channels and other media, including radio shows and newspapers [45, 46]. This road show proved to be memorable for senior managers in the districts.

### The role of the minister

Many senior managers mentioned the road shows, having attended at least one session to hear the Minister speak, noting it as a useful source of information to understand what NHI piloting and PHC-reengineering was about,

“*You don’t need healthy people going to a hospital; you need to remove those feet”* (SM1, Site 2).

*“There should be change, the way we do things; we need to change, especially at this time of NHI.” (SM1, Site 1).*

*“NHI is a vehicle for integration/platform for PHC centred care: an opportunity to integrate services as part of/embedded in the district, rather than stand alone.” (SM1, Site 2).*

The Minister of Health also personally participated in the dissemination of innovations within the districts, for example, addressing newly appointed hospital Chief Executive Officers in their induction programme (part of hospital reform) directly about PHC re-engineering and NHI piloting. This impressed one of the new CEOs, who had never experienced such lengthy face-to-face contact with the Minister before:

*“The Minister was with us, Monday to Friday. I have never seen something like that”* (SM3, site 2).

A *senior hospital manager in site 3* also mentioned the Minister’s role in communicating information and his focus on building management capacity in the CEO induction programme.^7^ The CEO noted he left the meeting feeling motivated to be a part of the PHC platform and noted that the Minister made it clear that a Hospital CEO’s job is to work in unison with the PHC platform:

*“Within [integrated planning] we said, let’s take primary health care, being the centre of our planning, because before, I was always concerned about we need extra beds for my hospital, that is what has been my issue. But now, I am not saying that, because I am saying, within two years, if the community health care worker programme is working well and we are going to communities where they are, it simply means the numbers of people that are going to end up in my hospital are going to go down. So, understand the pressure that is happening now, it is a temporary pressure.”* (SM3, site 2).

The Minister’s champion role did however have limits. In the early period, many private general practitioners did not attend the stakeholder events he hosted to promote contracting into public sector health clinics. Managers in two districts commented this was likely due to poor relationships between private general practitioners and the public sector due to previous late payments when doing sessional work in the public sector [47]. One senior manager commented that private GPs do not see the Minister as ‘their boss’ and felt this was disrespectful. In later months, sub district managers in Site 2 went door to door to GP offices to discuss the idea of contracting-in, in person, and this appeared to have worked better in starting a conversation. In site 3 there was a history of working with private GPs to improve access to care in the public service and this reform was seen as a continuation of existing practice.

### Broader dissemination and support from higher levels

While the Minister’s role was valued, it was felt better communication from the SA NDoH was needed on the role of the district in relation to specific innovations:

*"I think I have managed to sneak in to the National Department of Health to find somebody, Dr X, who is … dealing with the GP contracting. Now, I’m very much happy. I only managed to talk to that person, it was only last week, and she seemed to be a very cooperative lady … . You know, I’m going to be fine with that. Because I said to her, you know, "I do not know now what is it that I must do in relation to this? Initially, National said it’s a national prerogative to contract the GPs and we had to stop. A month later, an e-mail came: “Tell us how many GPs are willing to contract with you?”* (SM2, Site 1).

Some managers wanted information and evidence on where the new innovations came from and how other countries had implemented individual reforms, in order both to help make sense of the reforms for themselves and to support lesson sharing with staff (who expected managers to give direction and answer critical questions about implementation). One manager noted that while the National government saw them as implementers, staff saw them as managers and leaders.

Questions were also raised about what it meant to be a ‘pilot’ site and whether districts had the capacity to monitor and evaluate reforms,

“*I think if you are piloting, you need to be able to actually think outside the box yourself*.” (SM1, Site 3).

*“We haven’t been good in the Province [at monitoring and evaluation] … how we going to examine what all nine Provinces are doing in the pilot districts to determine this is what we want, and this is what we going to roll out, but I don’t know how.”* (SMP1, Site 2).

Participants felt that more effort could have been made by the SA NDOH to effectively communicate about the new range of new stakeholders entering the district, who often arrived unannounced.

*“I know that the Office of Standard Compliance [team] usually comes [to inspect our facilities] but we used to know when they are coming. [In contrast] You know National has contracted so many companies, areas, other programmes in relation to the NHI. You will be surprised that others, they don’t even show you an appointment or a contract with the Department of Health to say we have been appointed to do this. You would be just seeing them moving around and you will be just hearing by the manager, saying that there are people arriving in our institution. They are saying they want to check this and that and so on.”* (SM2, site 1).

Unannounced visits from Provincial or National supervisors were also felt to have a disruptive effect on the daily working of the managers as they then had to forego their own plans for the day.

All managers agreed that scheduling meetings (e.g. for the whole year) enabled managers to prepare accordingly. NHI coordinators in two districts valued scheduled meetings with National staff as they could air their frustrations and present problems, such as administrative constraints to spending money or other teething problems related to the service delivery innovations. Facility improvement teams^8^ were identified as good at scheduling meetings - staff turnover did however disrupt scheduling when a new incumbent chose not to follow existing schedules in one district.

### Innovations aligned to pre-existing local strategies

When new innovations were well aligned to local needs and existing strategies, they were more quickly diffused into the system. In the case of the ‘District Clinical Specialist team’ reform, for example:

“*They were able to extend an existing vision of getting specialists into the district through the District Clinical Specialist teams (DCSTs). The pre-existing provincial vision of a family physician-led team was well aligned to the National Plan.*” (SM5, site 2).

Regarding the management strengthening reform, some districts had an existing organisational culture of developing managers in the system and district managers welcomed a renewed focus on management and leadership strengthening;

Some staff ... “*they started off as pharmacy assistants; they died as deputy directors because of the growth through the system and actually managing talent to make sure that we develop them.*” (SM1, Site 2).

In district 3, the district manager felt that managers in the DMT had extensive training and that a focus on management training should be diffused downward to local level managers. There was, thus, general buy in for the idea of management training.

### Synergies between innovations

Actors involved in one innovation helped in diffusing and disseminating other innovations as they were introduced into the district, e.g. the district clinical specialist teams were at times inducting or supervising GPs in the roll out of GP contracting in public clinics and were also playing a role in consulting on and improving the referral system. New CEOs of hospitals who had been through induction and training via a hospital revitalisation programme were actively engaging in thinking through the role of the hospital as an integrated part of the PHC reengineering initiative.

Organisational learning was also taking place, the district developing an induction programme for GPs noted that the SA NDOH was learning from their development of the programme.

### NHI piloting coordinators in the district

Each NHI pilot district was required to appoint a district NHI coordinator to facilitate communication up and down with National, Provincial, district level and external stakeholders. Even though no formal position existed on an organogram, there was awareness of the role and NHI coordinators were in place in two of the three districts. In practice, the NHI District Coordinators were project-managing the NHI piloting initiatives and played a key role in spreading the message of NHI piloting in the district.

Being included as part of senior management made the NHI coordinators feel valued. In District 1, the NHI coordinator was identified from the existing district staff complement and assigned the additional management task of managing programmes in the district. Being from the district, he used his existing peer networks in the district as well as his new platform as a programme manager to diffuse and disseminate information:

“*So broadly, I am involved in such programmes. (…) You need to meet with key stakeholders (…) I am able to contact and to talk to these people, to make sure that we wean them towards getting, knowing, wanting to know this NHI [rather] than to have a negative attitude”* (SMS2, site 1).

In District 2, the NHI coordinator expressed gratitude at being mentored by a senior manager with years of management experience in the district as he was new:

*“You know, for me, I must say, luckily, I’ve got into the position where there was already somebody who had already laid the foundation …. So, I must say I view myself as being one of the lucky guys who came into a vehicle that was already moving. All that I had to do was to catch up with the speed at which it was moving”* (SMS2, Site 2).

As rules and guidance on the implementation of innovations changed over time, changes were not always effectively communicated downward from the National level to the NHI coordinators, which meant they were sometimes left confused on the way forward.

### Challenging implementation contexts

In two districts, district managers had limited decision space to spend money without the approval of their respective provincial governments. This limited manager control and ability to help quickly where needed. Vacancies and a shortage of managers meant that the burden of adopting and implementing new reforms fell on too few staff, which created anxiety for senior managers about the implications for the implementation of structures that would be needed should the full plans for National Health Insurance be implemented:

*“[Missing posts are] critical because when you look at where the country, the policy directives for National health Insurance specifically. Until such time that you've built management capacity, and the core point for the NHI is that you should have a very strong supply chain management process and also human resource management. Those are the parts that are lacking because with that district health authority that has to purchase services for the district, that’s where the problem comes in if you don’t have a full complement.*” (SM1, Site 2).

All districts suffered from some basic operational constraints, ranging from infrastructure challenges and shortage of equipment to data verification procedures:

*“We don’t have needles in theatre .... The money is there, but nobody is buying. They don’t know how to do it, how to procure.”* (SM1, site 1).

A manager in District 3 noted such constraints often meant they were non-compliant with national core standards,^9^ casting them as a poor performer. For example, not always having a general assistant in a facility meant they did not meet the cleanliness standard.

A provincial level manager in District 3 expressed concern over a lack of change management culture in public service systems, reflecting on long bureaucratic recruitment processes – “*3 months’ just to get a post advertised*” (SMP1, site 3), and about the huge amounts of information needed simply start the process of performance management for underperformers. He was concerned about the capacity of a “*passive*” public service to absorb creative and innovate changes.

Senior managers have to manage the daily functioning of the district while also trying to lead change through their staff, a senior manager noted;

*“I think the preparation for NHI relies heavily on innovation and in order to innovate properly, you need a stable system. This is an extremely unstable system, so you have got to innovate and stabilise at the same time, which I think adds a lot to the complexity of what we do.”* (SM3, Site 1).

### Contextual enablers

Even though initial challenges were evident, there was a strong sense that managers valued the additional funding which came with being an NHI pilot site, as it would help improve the context:

*“There are plans to rebuild three hospitals over the next four years with National. There are plans to rebuild sixteen clinics, eight of which National is doing, so I am getting a lot of support from national. Some of it, I didn’t ask for, but it’s kind of is in line with what is needed. So, I am not going to say “No, thank you.” … They are also doing maintenance on forty-five clinics. The sixteen clinics that are being built, they are coming with equipment and everything”* (SM3, site 1).

The innovations under the banner of NHI piloting also benefited from the history of PHC in South Africa, PHC staff already working in the system and routine meetings and discussion spaces at the Provincial and health district level for PHC. Staff discussed PHC and NHI piloting matters together. For example, the NHI coordinator in District 2 was chairing the district level PHC forum, in which he engaged all sub district focal people within the health service, such as sub district managers, to assist in NHI roll out in these spaces. In Provincial PHC spaces, the NHI coordinator in district 2 said there were times he felt overwhelmed by questions on PHC as he was only in control of the funding for PHC under the NHI conditional grant; he thus always attended provincial PHC re-engineering task team meetings with the district PHC manager who understood and managed PHC under the normal provincial budget allocation.

Existing inter-sectoral platforms at the local level, like the Integrated Development Planning processes at local government, were used to disseminate information to a range of local government departments and community councils and structures in district 1.

*“We are finished with the mayors at this level. Now, we are going to each individual in the municipalities to meet with the councillors there. We make the presentation that says what the NHI is. I think that is just key; at the same time, how is it going to be implemented? Then how far we have gone in this five-year pilot phase, because other people are thinking we are supposed to be implementing this [the financial reform] now. They are not aware that we are talking of a preparatory stage [the first years of system strengthening]”* (SM2, Site 1).

An NHI district coordinator conveyed the importance of promoting an understanding that NHI piloting reforms were connected to existing National mandates in the district, such as the ‘10 Point Plan for 2009-2014’ - a list of ten priorities which included overhauling the health system and implementing national health insurance [48]. Quality improvements were also an extension of the existing ‘National Core Standards for Health Establishments in South Africa’, an existing benchmark of quality of care against which service delivery is monitored [49]:

*“What is in the ten-point plan for instance, … one of the points that appear there is the implementation of the NHI. You know just for people to understand where we come from with NHI, that it did not just fall from nowhere. You know, it was in the plan, and say this is what we want to do”* (SM2, Site 2).

Existing local horizontal networks were also useful in leveraging resources for individual NHI piloting reforms. In District 1, it was identified that the new Community Health Worker (CHW) programme could benefit from environmental officers already working there. In District 2, efforts were underway to set up communication structures between CHWs and community-based planners from the municipality as both actors made home visits. In District 1, managers were engaging a staff member from the local university to mentor the new District Clinical Specialist team, while the District Manager in District 2 were leveraging private sector resources for health promotion activities to support PHC Re-engineering. These existing structures and relationships thus allowed for a natural diffusion of ideas and interests if identified as opportunities by managers.

District NHI coordinators also worked through new vertical structures set up by the National and Provincial governments including a Provincial NHI task team made up of senior managers in charge of different programmes at the Provincial level to whom NHI district coordinators reported. Quarterly NHI financial and progress reports in the district had to be signed off by the Provincial Head of Department and forwarded to the National Department from there – initially there were some trust concerns between district actors and provincial actors. Managers in districts were not able to identify all the new NHI structures as they were only starting to engage with some structures toward the end of the study. We did however observe that Provincial NHI coordinators were starting to visit the district more often and taking on shared responsibility in project managing the NHI piloting portfolio in the districts (one had already started playing a major role).

## Discussion

In the period examined, South Africa did make progress in implementing NHI-linked reforms, highlighting that diffusion and dissemination are ongoing processes that run before and parallel to implementation. The case offers some lessons for the dissemination and diffusion of innovations in ways that are helpful for health managers at the coal face.

Dissemination strategies and the concept of natural diffusion identified in the Greenhalgh et al. [21] model are used to frame key learnings on disseminating and diffusing reforms.

The existing local context presented both challenges and opportunities for these processes. Key strategies that supported them included the use of champions and the use of new and existing horizontal and vertical networks. Well or poorly planned communication strategies and the availability of support structures for managers also affected dissemination. Managers were not only subject to dissemination strategies but also played critical sense making and sense giving roles as part of the dissemination and diffusion of innovations [18, 50]. They also played boundary spanning roles connecting with those outside the health system to leverage support. We did not identify any opinion leaders or homophily playing a role in the processes examined.

### Innovation system fit

Innovation system fit represents the interplay between the innovation and the context within which it is embedded [21]. Challenges found across the SA DHS mirror contextual challenges facing the adoption of new reforms in the three district NHI pilot sites. These included a continued shortage of managerial capacity partly due to vacancies, infrastructure shortages in facilities, limited staff to drive and implement new reforms, poor relationships with the private health sector and in some cases, poor relationships between provincial and local governments [28, 33].

Opportunities for diffusion included similarities between new NHI innovation ideas and existing practices and policies. In one district, as a system of doctors visiting public clinics was already in place, the new model of GP contracting was much easier to absorb. Existing PHC provincial and local networks were also purposefully used to keep managers informed of updates from the SA NDoH and for reporting upward on progress of NHI. Institutions thus provide the ‘scaffolding’ that networks are structured around, and ideas are spread through new and old networks [51]. Similarly, policy legacies including administrative capacity, positive experiences with an innovation in the past, and a well-established research environment that supported policy learning over time, proved beneficial in the reintroduction of Malaria home case management in Burkina Faso [51].

The senior managers embedded in the local South African context were also able to stimulate synergies between different innovations. For example, the District Clinical Specialist Teams (DCSTs) developed an induction programme for newly contracted GPs and provided clinical oversight of GPs – thus better enabling innovation system fit of the GP contracting reform. The introduction of new teams (or restructuring of existing teams) and posts in the districts are thus both part of the NHI piloting innovation and over time can become a vehicle for its dissemination and diffusion. This experience supports a much longer view of dissemination and diffusion over time as system components combine to produce unexpected and novel outcomes [52].

### The use of champions

Reform champions can be used to garner support for innovations from a range of actors. The Minister, by participating in the roadshow and in his personal capacity, inspired managers; Greenhalgh et al. [21] identify a champion who can garner support from other individuals as a *transformational leader*. The Minister’s message of equity resonated with senior managers and addressed a shared value - ideas and discursive frames shape how people think about new reforms and are thus important dissemination tools [53] Political elites actively showing support have also been an important factor in garnering support at the local level during early stage implementation of community health workers programmes in South Africa [54] and Greenhalgh et al. [21] found that innovations are more likely to be adopted when they resonate with the values and beliefs of those expected to adopt and implement them. While political elites supporting population health as a priority can facilitate the mobilisation of financial and human resources and create political will at the local level, other important factors that are key to successful diffusion include continuity and consistency in policies over time and a stable bureaucracy with competent managers who have sufficient power to manage change [55]. Resources, early communication and managerial support are equally important to successfully giving effect to values on the ground [56, 57].

The SA NDoH requiring the appointment of an NHI coordinator usefully assisted the dissemination and diffusion on innovations in the DHS. The coordinator played the key champion role of *network facilitator,* an individual who develops cross functional networks and coalitions across the organisation, as well as a project management role. They actively participated in existing and new PHC and NHI networks across all three tiers of government, as well as, at the local inter- sectoral level playing a boundary spanning role,^10^ to gather and report information and at times leverage resources for implementation. The ‘enablement of knowledge sharing via internal and external networks’ is a key system antecedent for the diffusion, dissemination and implementation of innovations [21]. A NHI coordinator, in his champion role benefited from having worked in the district previously as he could leverage an existing set of existing peer and social networks, “the pattern of friendship, advice, communication and support which exists among members of a social system” has been found to be a dominant mechanism for diffusion [21, 58]. Acceptance by the district senior management team, mentorship to help understand the DHS and acknowledgement of NHI piloting as a major project in the district also aided this champion role. A need was identified for a support person at National level whom the NHI coordinator could contact for early information and clarity on processes related to specific reforms.

### Planned dissemination strategies and the use of networks

#### Good communication

The road show and the introduction of the Monitoring & Evaluation team (a vertical technical support team) by the National government both appear to be planned dissemination strategies that facilitated diffusion and dissemination. Key success factors of the M & E vertical network included a willingness to negotiate with management in district offices on priorities in the business plan, being responsive to communication requests, feeding back information to national government in a timely fashion and having the authority to make quick decisions allayed initial concerns surrounding the conditional grant and the development of the business plan. The implementation of a private medicine retailer programme for Malaria in Kisii, Kenya similarly benefitted from a technical team that understood the district context, previous work experience in the district and specific operational and strategic thinking experience related specifically to Malaria, which helped identify challenges prospectively [59]. A memorandum of understanding which laid out a clear set of principles for engagement had also been set up. Other skills required of technical support teams include a unifying vision, an understanding of the local context and its capacities, be well connected, and have coalition building and technical skills [60].

Greenhalgh et al. [21] note that the use of formal planned dissemination strategies that target particular audiences with appropriate messaging and appropriate communication channels can facilitate innovation diffusion [21].

#### Areas where there is a need for more well-planned dissemination strategies

Senior managers felt that more specific dissemination of information on where innovations came from, what evidence supported the specific innovations and practical success stories from other countries was needed to help them ‘sense give’ to their staff; and a lack of information dissemination about new stakeholders entering the district was also identified as a challenge by the senior managers. in another South African experience, the roll out of mental health policy guidelines also suffered from the lack of development of a formal dissemination process and a lack of advocacy to lower levels of the system about the nature of the new policy [61]. Large scale reform toward UHC in Colombia suffered from limited dissemination of information on regulations and rules within the system and from limited information sharing with users which affected roll out [62].

Well planned dissemination strategies about new reforms are important processes to help managers in districts roll out reforms as they have to engage in ‘sensemaking’ for themselves and then engage in ‘sense giving’ to staff [50, 63]. McIntyre and Klugman [17] write that managers need to receive timely communication about new policies, so that they in turn can adequately communicate with and motivate their staff, communication should also be collaborative [56].

#### Recommendations for planned dissemination strategies and natural diffusion

While planning for dissemination and communication are critical components for implementation success, Barrett and Fudge [24] caution that we should not simply see communication as a tool by which those at the top to coordinate the actions of those below. Determining the right amount of top down national guidance with the right amount of bottom up local flexibility in adoption and implementation will always be a balancing act [64]. With a focus on the ‘best’ or ‘standardised’ way of doing things organisations are losing out on the benefits of innovation and creativity [24]. Plsek & Wilson [65] recommend developing a set of minimum specifications or simple rules developed through dialogue by relevant stakeholders involved in the process of change – the minimum specifications provide a broad framework within which to work, should be direction pointing, show boundaries, identify resources and set permissions. The specifications will not be perfect, will evolve over time and are not ‘standards’ – they lay the foundation for creativity. In the scale up of antiretroviral treatment (2005–2007) across three provinces in South Africa, Schneider et al. [66] found that the province that rejected a standardised rigid approach and opted for the development of simple rules over time through joint learning, the use of local tacit knowledge and partnership with others, was able to improve treatment coverage successfully. There is a growing body of research highlighting the importance of ‘emergent and voluntary coordination, collaboration and partnerships’ in promoting adaption and learning over time [67].

### Limitations

The findings reflect the bottom up experiences of senior managers in districts - they therefore only include information on dissemination and diffusion efforts from their perspective. The National government may have implemented a range of other dissemination efforts that are perhaps undocumented or not mentioned by participants and therefore go beyond what is addressed in this article. Senior managers only represent one cadre working at the district level, facility level staff may have other views.

## Conclusion

This study adds to our understanding of the experiences of local level managers who are at the receiving end of top down UHC reforms. The early stages of the dissemination of innovations can cause anxiety for managers as they must make sense of new ideas and practices for themselves and for their staff in challenging contexts, sometimes with limited information on the innovations and a lack of clarity on key processes. Senior South African health managers in districts do however believe in the need for change and use tacit knowledge, play boundary spanning roles and leverage networks to further diffuse reforms, promote adoption and get innovations implemented. Well planned dissemination strategies that include early communication, the use of feedback loops, the setting up of communication support structures, the use of champions as well as the use of new and existing networks can help managers to make sense of and lead change. As countries move to institutionalise a range of technical proposals and solutions to achieve UHC, the importance of early, well planned and continuous dissemination strategies that facilitate processes of adoption and implementation should not be forgotten.

## Additional file

Additional file 1: Innovations in NHI pilot districts. A description of some of the key innovations being disseminated (or planned to be) into the NHI pilot districts in 2012 as part of the NHI piloting and PHC re-engineering process. (DOCX 20 kb)

## Abbreviations

LMICs: Low- and Middle-Income Countries
M & E team: Monitoring and evaluation team
NHI: National Health Insurance
SA NDoH: South African National Department of Health
UHC: Universal Health Coverage

## Footnotes

1 NHI pilot site: The overall purpose was to pilot reforms to strengthen the district health system in eleven selected health districts in South Africa,

2 South Africa currently promotes a PHC approach to healthcare, ideally a comprehensive set of health services including preventive and promotive services, is community based and engages multiple sectors embedded in a social understanding of the community. PHC re-engineering included ward-based outreach teams, a renewed focus on school health services and the introduction of clinical specialist teams into the district health system.

3 An additional table provides a description of some of the innovations that were being implemented in the NHI pilot sites (see Additional file 1)

4 An additional table provides a description of some of the innovations that were being implemented in the NHI pilot sites (see Additional file 1)

5 Traditional budgetary resources.

6 Based on information from interviews, the M & E team was a group of 3–5 individuals from the National Department of Health who were sent as a technical team to provide support and monitor progress in the districts.

7 An additional table provides a description of some of the innovations that were being implemented in the NHI pilot sites (see Additional file 1)

8 An additional table provides a description of some of the innovations that were being implemented in the NHI pilot sites (see Additional file 1)

9 Quality assurance measures developed by the National Department of Health against which service delivery by health establishments can be assessed (http://phisa.co.za/wp-content/uploads/2019/01/National-Core-Standards.pdf)

10 “those who have significant social ties inside and outside the organisation and are able and willing to link the organisation to the outside world with regards to a particular innovation”
