# Supplementary material for: A qualitative study of the dissemination and diffusion of innovations: bottom up experiences of senior managers in three health districts in South Africa
Source: Int J Equity Health. 2019 Mar 29;18:53. doi: 10.1186/s12939-019-0952-z (PMC6441208; doi:10.1186/s12939-019-0952-z)
Supplement: Supplementary file 1 — Innovations in NHI pilot districts. A description of some of the key innovations being disseminated (or planned to be) into the NHI pilot districts in 2012 as part of the NHI piloting and PHC re-engineering process. (DOCX 20 kb) [file 12939_2019_952_MOESM1_ESM.docx]

**Additional file 1**

**Title of Table:** Innovations in NHI pilot districts

**Description of table:** A description of some of the key innovations being disseminated (or planned to be) into the NHI pilot districts in 2012 as part of the NHI piloting and PHC re-engineering process^[[1]](#footnote-1)^.

| GP contracting | The contacting in of private general practitioners to work in public facilities in communities that are currently under-served, to provide services that enhance the current PHC model, for example personal curative services, health promotion and preventative care, ensuring effective chronic disease management and PHC team capacity building etc. (1) |
| --- | --- |
| District Clinical Specialist Teams | “In order to address high levels of maternal and child mortality and to improve health outcomes an integrated team of specialists will be based in the districts. The specialities will include: a principal obstetrician and gynaecologist; a principal paediatrician; a family physician; a principal anaesthetist; a principal midwife and a principle primary health care professional nurse. Others will be added over time as the need arises. The role of these teams will be to provide clinical support and oversight particularly in those districts with a high disease burden” (2). |
| Management strengthening | The Minister has recognised the important role of health managers and leaders who are able to manage and lead complex health systems. A range of interventions were planned under this intervention area, two examples include:   - The designation of hospitals and a policy on the management of hospitals: The clear designation of hospitals allowed for clarity on the competencies required of Hospital CEOs and the decentralisation of management and development of accountability frameworks. A competency assessment of existing hospital CEOs was completed and where required, new hospital CEOs were recruited (1). - A future idea was also the establishment of a South African leadership and management academy for those in the health system (3) |
| School based PHC health teams | “School health services will be delivered by a team that is headed by a professional nurse. The services will include health promotion, prevention and curative health services that address the health needs of school-going children, including those children who have missed the opportunity to access services such as child immunization services during their pre-school years. School health is an integral part of the comprehensive package of primary health care services that must be delivered to every school in the district” (2). |
| Municipal ward based PHC teams. | “A team of PHC agents will be deployed in every municipal ward. At least 10 people will be deployed per ward. Each team will benefit from a health professional depending on availability. Each member of the team will be allocated a certain number of families. The teams will collectively facilitate community involvement and participation in identifying health problems and behaviours that place individuals at risk of disease or injury; vulnerable individuals and groups; and implementing appropriate interventions from the service package to address the behaviours or health problems” (2). |
| Public health facility audits | This included an audit of all public clinics, community centres and district, regional, specialised and tertiary hospitals in all nine Provinces in South Africa. (1) |
| Facility Improvement teams | Primary focus on improving quality in facilities. Teams were trained in quality improvement and were established to address the findings of the public health facility audit and to strengthen the supervision of services. The teams supported the development of quality improvement plans for health facilities in the NHI pilot districts (1) |

**References**

1. Matsoso MP, Fryatt R. National health Insurance: The first 18 months. In: Padarath A, English R, editors. South African Health Review 2012/2013. Durban: Health systems Trust; 2013. p. 21-33.

2. South Africa National Department of Health [SA NDoH]. National Health Insurance in South Africa: Policy paper No. 657. South Africa: SA NDoH; 12 August 2011.

3. Bateman C. Health leadership training academy tackles worst first. SAMJ: South African Medical Journal. 2013;103(10):707-8.

1. This is not a comprehensive list of reforms taking place in the country that are relevant to the NHI piloting process, a range of policy processes related to quality, human resources and information management were also in process. It is provided to give a sense of some of the reforms, our larger project specifically focused on the first five in the table. [↑](#footnote-ref-1)
